# Hepatocyte growth factor is necessary for efficient outgrowth of injured peripheral axons in *in vitro* culture system and *in vivo* nerve crush mouse model

**DOI:** 10.1016/j.bbrep.2021.100973

**Published:** 2021-03-03

**Authors:** Nayeon Lee, Sang Hwan Lee, Junghun Lee, Mi-Young Lee, Jaegook Lim, Subin Kim, Sunyoung Kim

**Affiliations:** a School of Biological Sciences, Seoul National University, Seoul, 08826, South Korea; b Division of Gene Therapy, Helixmith Co Ltd, Seoul, 07794, South Korea

**Keywords:** HGF, c-met, Sensory neuron, Neurite outgrowth, Axon regeneration, Peripheral neuropathy

## Abstract

Hepatocyte growth factor (HGF) is a neurotrophic factor and its role in peripheral nerves has been relatively unknown. In this study, biological functions of HGF and its receptor c-met have been investigated in the context of regeneration of damaged peripheral nerves. Axotomy of the peripheral branch of sensory neurons from embryonic dorsal root ganglia (DRG) resulted in the increased protein levels of HGF and phosphorylated c-met. When the neuronal cultures were treated with a pharmacological inhibitor of c-met, PHA665752, the length of axotomy-induced outgrowth of neurite was significantly reduced. On the other hand, the addition of recombinant HGF proteins to the neuronal culture facilitated axon outgrowth. In the nerve crush mouse model, the protein level of HGF was increased around the injury site by almost 5.5-fold at 24 h post injury compared to control mice and was maintained at elevated levels for another 6 days. The amount of phosphorylated c-met receptor in sciatic nerve was also observed to be higher than control mice. When PHA665752 was locally applied to the injury site of sciatic nerve, axon outgrowth and injury mediated induction of cJun protein were effectively inhibited, indicating the functional involvement of HGF/c-met pathway in the nerve regeneration process. When extra HGF was exogenously provided by intramuscular injection of plasmid DNA expressing HGF, axon outgrowth from damaged sciatic nerve and cJun expression level were enhanced. Taken together, these results suggested that HGF/c-met pathway plays important roles in axon outgrowth by directly interacting with sensory neurons and thus HGF might be a useful tool for developing therapeutics for peripheral neuropathy.

## Highlights

• In *in vitro* primary eDRGs, axotomy-induced HGF/c-met pathway enhanced the neurite outgrowth process.
• Nerve injury induced the expression of HGF, consequently leading to the activation of c-met in peripheral axons.
• HGF/c-met pathway played an important role in the regeneration process of injured peripheral nerves.
• Additional supply of HGF, in the form of plasmid DNA, enhanced the regeneration of damaged peripheral nerves.

## Abbreviations

**HGF**: hepatocyte growth factor
**DRG**: dorsal root ganglion
**RAG**: regeneration associated gene
**eDRG**: embryonic DRG

## 1 Introduction

The regeneration process of peripheral nerve is initiated immediately after nerve injury [1]. Initially, two types of signaling pathways are activated to induce the formation of the growth cone. Calcium rapidly enters the cell, and is then transported to the DRG, acting as an injury signal [2,3]. Later, additional signaling pathways involving ERK, DLK, JNK, and STAT3 are relayed to the DRG to induce the expression of regeneration-associated genes (RAGs) including ATF3, cJun, STAT3, galanin, and GAP43 [4]. A variety of biological reactions and activities follow the expression of these genes, including the retro/anterograde transport of protein cargos, rearrangement of cytoskeletons, and translation of axonal mRNAs to promote the axon outgrowth process [5].

Hepatocyte growth factor (HGF) plays an important role in survival, proliferation, and migration of various cell types by activating c-met receptor [6]. In the context of the peripheral nerve, the HGF/c-met pathway is known to induce neurite outgrowth of sensory neuron during the developmental stage. HGF has been shown to increase the length of neurite of cultured embryonic DRG neurons in an NGF-dependent manner. HGF also promotes the survival of DRG neurons. In addition, mice containing mutations in c-met receptor show reduced innervation of sensory neurons to the skin. In embryonic motor neurons, HGF enhances the survival and proper innervation of neurons to the target tissue. HGF also facilitates the neurite outgrowth of embryonic sympathetic neurons [7,8].

HGF also interacts with c-met receptors present in Schwann cells located in distal regions of the injury site. HGF induces the proliferation and migration of cultured Schwann cells and upregulates the expression of GDNF and LIF by activating the Erk pathway. Finally, injury-induced HGF/c-met pathway enhances the re-myelination of Schwann cells, thereby promoting the process of peripheral nerve regeneration [9,10].

In addition, some serine proteases such as urokinase plasminogen activator (uPA) and tissue-type plasminogen activator (tPA) are highly expressed by nerve crush injury. Single chain HGF, known as pro-form, is cleaved by serine proteases such as uPA and tPA into α-chain (containing four kringle domains) and β-chain (containing serine protease-like structure). These chains are linked by a disulfide bond to become a mature form. This mature HGF is a biologically active form and shows a high affinity for c-met receptor. Peripheral nerve injury upregulates the expression of uPA and tPA. In addition, mice lacking uPA and tPA showed reduced recovery of injured nerves. Thus, the peripheral nerve injury might elicit the expression of mature-form HGF [11,12]. However, it has not been clear whether HGF also interacts directly with peripheral neurons to exert biological effects on axon outgrowth.

In this study, the role of HGF/c-met pathway was investigated in damaged peripheral neurons. In axotomized embryonic DRG cultures, the level of both HGF and phosphorylated c-met was highly increased. Similar observations were made in the nerve crush mouse model. The inhibition of c-met receptor by PHA665752 hindered the outgrowth of axons and injury-mediated induction of cJun protein. When additional HGF was exogenously provided, axon outgrowth was facilitated as measured by nerve pinch test. Our results strongly suggest that HGF is an important part of the axon regeneration process in injured nerves by directly interacting with neurons.

## 2 Materials and methods

### 2.1 Animals

All experimental protocols were performed under the guidelines of the Seoul National University Institutional Animal Care and Use Committee (Permission No. SNU-170426-1-3). Eight-week-old male C57BL/6 mice were purchased from Orient Bio Inc. (Gyeonggi-do, Korea). Mice were housed at 22 °C with a 12-h light-dark cycle, given access to food, and provided water ad libitum. To induce peripheral nerve injury, sciatic nerve crush was performed as described previously [13]. Animals were anesthetized with isoflurane and the sciatic nerve was exposed by making a small incision in the skin and muscle. The exposed nerve was then crushed for 15 s using fine hemostatic forceps (FST, British Columbia, Canada). Incisions were sutured and animals were monitored for their recovery.

For nerve pinch tests, mice were mildly anesthetized with low concentration of isoflurane. The sciatic nerve was exposed and pinched from distal to proximal direction until a reflex response of the hindlimb was observed. The distance between the pinched site where animals showed the reflex and injury site was measured.

After nerve crush injury, plasmid DNAs were injected to the bicep femoris muscle with 200 μg/head. For PHA665752 (Tocris, Bristol, United Kingdom) administration, surgifoam was soaked with DMSO solution containing 25 mg/kg of PHA665752 and delivered to the local injury site.

### 2.2 Primary embryonic DRG culture

DRGs from E13.5 ICR mice were collected, following trypsinization with 0.25% Trypsin/EDTA (Gibco, MA, United States) for 22 min. Each well coated with PDL/laminim (Thermofisher, MA, United States) was spotted with 5–7 DRGs and dried for 18 min. Cells were then cultured with 500 μL of Neurobasal media (Gibco) including 25 ng/mL of the recombinant NGF protein (R&D, MN, United States), 1:50 of B27 (Gibco), 1:100 of Penicillin/Streptomycin (Gibco), 1:100 of Glutamax (Gibco), and 1:200 of fluorodeoxyuridine (FdU) (Sigma, MO, United States). A week after spotting, axotomy was performed with a chisel (FST), followed by SCG10 staining after 12–40 h to measure regenerated axons.

### 2.3 Immunocytochemistry (ICC)

Cultured cells were fixed in 10% formalin for 15 min at room temperature. Cells were then washed three times with PBS followed by incubation with the blocking solution (10% FBS with 0.2% Triton X-100 in PBS) at room temperature for 1 h. Primary antibodies specific to SCG10 (Novus, CO, United States, #NBP1-49461) in a 1:500 dilution, phosphorylated c-met (Cell Signaling Technology, MA, United States, #8218) in a 1:100 dilution, and βIII tubulin (BioLegend, CA, United States, #801202) in a 1:500 dilution were used at 4 °C for overnight. After three times of PBS washing, cells were incubated with secondary antibodies specific to rabbit IgG (Invitrogen, MA, United States, #A-21206) in a 1:500 dilution and mouse IgG (Invitrogen, #A-21424) in a 1:500 dilution at room temperature for 2 h. Immunofluorescence was measured using the IN Cell Analyzer 2000 (GE Healthcare, MA, United States) and the intensity was analyzed using Image J.

### 2.4 Immunohistochemistry (IHC)

Sciatic nerves were fixed in 10% formalin at 4 °C for overnight incubation. After washing with PBS, the tissues were sequentially immersed in 15% sucrose at 4 °C for 8 h and then 30% sucrose at 4 °C for overnight incubation followed by cryopreservation in the OCT compound (Sakura Tissue Tek, CA, United States). Samples were then cryosectioned with the thickness of 12 μm. After an hour of incubation in blocking solution (2% BSA with 0.1% Triton X-100 in PBS), samples were treated with primary antibody specific to SCG10 (Novus, #NBP1-49461) in a 1:500 dilution, phosphorylated c-met (Cell Signaling Technology, #8218) in a 1:100 dilution, and βIII tubulin (BioLegend, #801202) in a 1:500 dilution at 4 °C for overnight incubation. After three times of washing with PBS, samples were then incubated with secondary antibodies specific to rabbit IgG (Invitrogen, #A-21206) in a 1:500 dilution or mouse IgG (Invitrogen, #A-21424) in a 1:500 dilution at room temperature for 2 h. Immunofluorescence was measured with the IN Cell Analyzer 2000 (GE Healthcare) after mounting with DAPI (Vectashield, PA, United States).

### 2.5 Elisa

ELISAs were performed according to the manufacturer's protocol. Briefly, after 48 h of axotomy, supernatants were harvested followed by Mouse/Rat HGF quantikine ELISA kit (R&D). After 1, 3, and 7 days of injury, sciatic nerves were lysed using a RIPA Buffer (Cell Signaling Technology) with Protease/Phosphatase Inhibitor Cocktail (Cell Signaling Technology). Fifty microliters of protein lysate were loaded in a Mouse/Rat HGF quantikine ELISA kit (R&D) and incubated at room temperature for 2 h. After five times of washing with 400 μL of Wash buffer, wells were incubated with 100 μL of Mouse/Rat HGF Conjugate at room temperature for 2 h. After five times of washing with 400 μL of Wash buffer, wells were incubated with 100 μL of substrate at room temperature for 30 min. Then, wells were treated with 100 μL of stop solution and determined the optical density at 450 nm and 550 nm.

### 2.6 Western blot

Electrophoresis was performed with 20 μg of protein lysate loaded in a 4–12% Bis Tris gradient gel (Invitrogen) for 1 h. The gel was then transferred to PVDF membranes (GE Healthcare) for 1 h. Membranes were blocked with Blocker Casein in PBS (Thermofisher) at room temperature for 45 min. Membranes were incubated with primary antibodies specific to c-met (Sigma, #SAB4300599) in a 1:500 dilution, phosphorylated c-met (Cell Signaling Technology, #8218) in a 1:250 dilution, cJun (Cell Signaling Technology, #9165) in a 1:500 dilution, and GAPDH (Cell Signaling Technology, #3683) in a 1:500 dilution in a blocking solution at 4 °C for overnight incubation. After three times of washing with TBST (Invitrogen), membranes were incubated with secondary antibody specific to rabbit IgG (Cell Signaling Technology, #7074) in a 1:5000 dilution in a blocking solution at room temperature for 1 h and washed three times with TBST. Membranes were developed with 200 μL of Super Signal West Femto Maximum Sensitivity Substrate (Thermofisher) for 1 min and signals were visualized with ImageQuant Las 4000 (GE Healthcare).

### 2.7 Statistical analysis

Data are presented as the mean ± standard deviation (SD) or mean ± standard error of the mean (SEM). Statistical significance was assessed using unpaired *t*-test or one-way ANOVA followed by Bonferroni's multiple comparison testing. *p < 0.05; **p < 0.01; ***p < 0.001.

## 3 Results

### 3.1 Axotomy induces the expression of HGF in primary embryonic DRG (eDRG)

**Fig. 1:**
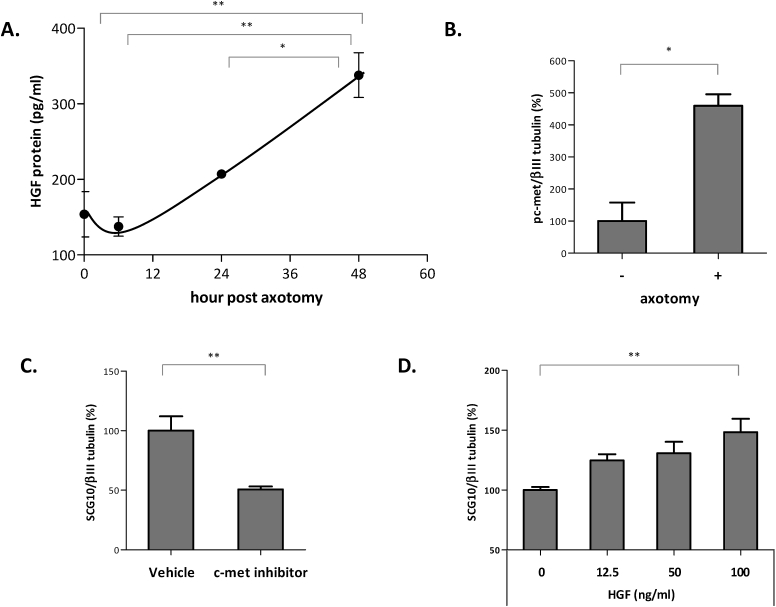
Activation of HGF/c-met pathway by axotomy in cultured eDRGs. A) Induction of HGF proteins by axotomy. Cultured eDRGs were axotomized. After 6, 24, and 48 h, the protein level of HGF in the culture supernatants was measured using ELISA specific for murine HGF. Values are shown as mean ± SD. For statistical analysis, one-way ANOVA followed by Bonferroni's multiple comparison testing was performed. *p < 0.05; **p < 0.01. B) Phosphorylation of c-met receptors by axotomy. Cultured eDRGs were axotomized and subjected to IHC using antibody specific to phosphorylated c-met. Values are presented as mean ± SD. Data was analyzed by unpaired *t*-test. *p < 0.05. C) Effect of c-met inhibitor, PHA665752, on neurite outgrowth. Cultured eDRGs were axotomized and treated with 1 μM of PHA665752 and then stained with SCG10 and βIII tubulin. The intensity of SCG10-positive axons in distal region was counted and summarized. Values are indicated as mean ± SD. Unpaired *t*-test was performed for statistical analysis. **p < 0.01. D) Effects of addition of recombinant HGF protein on neurite outgrowth. Cultured eDRGs were axotomized and treated with various concentrations of HGF recombinant proteins. Cells were stained with SCG10 and βIII tubulin. Intensity of SCG10-positive axons in distal region was quantified. Values are presented as mean ± SD. One-way ANOVA was performed, followed by Bonferroni's multiple comparison testing. **p < 0.01.

It has been reported that HGF/c-met pathway plays important roles in the development of sensory neurons [7,8], however, its role in injured peripheral nerve has largely been unknown. To investigate the involvement of HGF in damaged peripheral neurons, the level of HGF was first examined in embryonic DRGs. Embryonic DRG produced approximately 160 pg/mL of HGF protein, and its level was increased by 2.2-fold when axotomized (Fig. 1A). When axotomized, the number of cells positive for phosphorylated c-met was highly increased compared to control group, indicating that c-met was activated in injured neurons (Fig. 1B). Taken together, these data indicated that nerve injury increased the expression level of HGF and activated c-met receptor.

To test if HGF/c-met pathway is involved in axotomy-induced axon outgrowth, axotomized primary eDRGs were treated with PHA665752, a pharmacological agent that specifically inhibits activation of c-met receptor. The level of axon outgrowth was assessed by staining with an antibody to SCG10 protein, a marker specific for regenerating axons [14]. One day after axotomy, the sprouting pattern of SCG10-positive axons was readily observed. When treated with PHA665752, the number of SCG10-positive axons was decreased by 50%, suggesting that the blockade of c-met receptor signaling hindered the axon outgrowth program (Fig. 1C).

The additional supply of HGF protein to the neuronal cultures was then examined for further possible enhancement of axon regeneration. As shown in Fig. 1D, the number of SCG10-positive axons increased in a dose-dependent manner by up to 48% at the 100 ng/mL dose. These results suggested that HGF/c-met pathway was indeed involved in the regeneration of injured DRG axons and that exogenously added HGF protein could enhance this process.

### 3.2 Peripheral nerve injury induces the expression of HGF and activation of c-met in the nerve crush mouse model

**Fig. 2:**
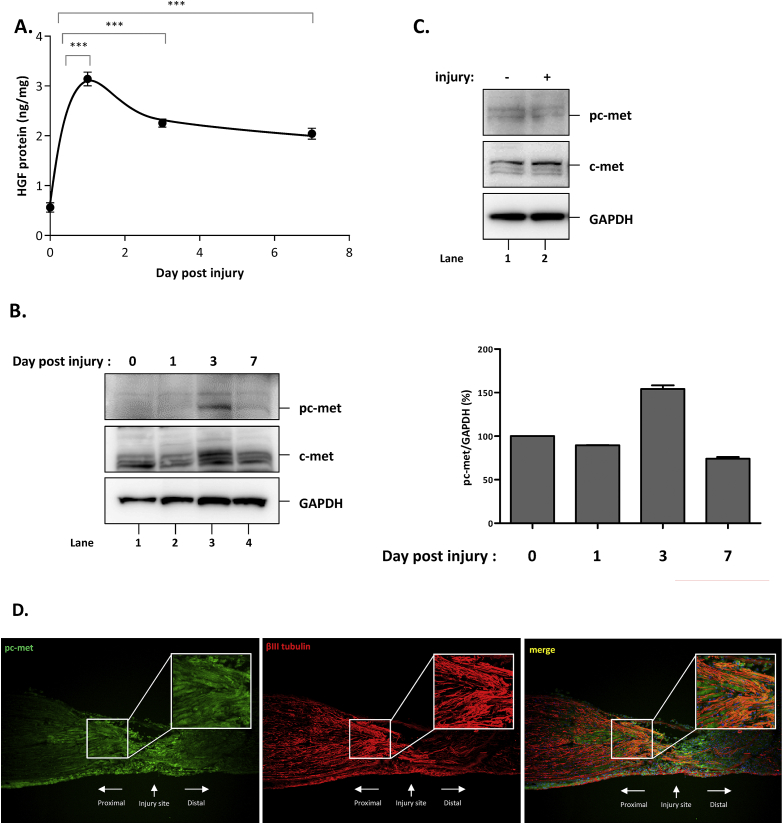
Effect of peripheral nerve injury on HGF and c-met. A) Increased amount of HGF proteins by sciatic nerve crush injury. Nerves from proximal to distal region were collected 0, 1, 3, and 7 days after injury followed by ELISA specific for murine HGF. Values are shown as mean ± SEM. For statistical analysis, data was analyzed by one-way ANOVA followed by Bonferroni's multiple comparison testing. ***p < 0.001. B) Phosphorylation of c-met receptor by nerve crush injury. 1, 3, and 7 days after injury, nerves from proximal to distal region were harvested. Proteins were prepared and subjected to Western blot analysis using antibodies specific to phosphorylated c-met, c-met, and GAPDH. C) Phosphorylation of c-met receptor on DRGs. DRGs were harvested 3 days after injury and proteins were prepared followed by Western blot using antibodies specific to phosphorylated c-met, c-met, and GAPDH. D) Phosphorylated c-met receptors localized in βIII tubulin positive axons. 3 days after injury, nerves were subjected to IHC using antibodies specific to βIII tubulin (in red) and phosphorylated c-met (in green). (For interpretation of the references to colour in this figure legend, the reader is referred to the Web version of this article.)

Involvement of HGF/c-met pathway in axon outgrowth was further tested in the nerve crush mouse model, which is widely used in the study of peripheral neuropathy-related axon regeneration program [15]. First, the expression kinetics of the HGF protein were examined. The level of HGF protein was highly increased by 5.6-fold at day 1 post injury and then gradually decreased through day 7 in the injured sciatic nerves (Fig. 2A). The level of phosphorylated c-met was also measured in the sciatic nerve and DRG. The level of phosphorylated c-met was increased by 1.5-fold at day 3 and eventually reduced to the basal level by day 7 (Fig. 2B). In DRG, no significant change in c-met phosphorylation was observed, indicating that nerve injury-mediated c-met activation might be restricted to the sciatic nerve (Fig. 2C).

Activation of c-met receptors was also analyzed by immunohistochemistry. A large number of cells positive for phosphorylated c-met (green) were observed in the proximal, distal, and injury sites of the damaged nerve. Many of these cells were merged with those positive for βIII tubulin-positive axons in the proximal region of the sciatic nerve, indicating that c-met was activated in neurons (Fig. 2D). Taken together, these data suggested that nerve injury might induce the expression of HGF in the sciatic nerve, consequently leading to the activation of c-met receptor in proximal peripheral axons.

### 3.3 The HGF/c-met pathway is involved in the peripheral axon outgrowth in the nerve crush mouse model

**Fig. 3:**
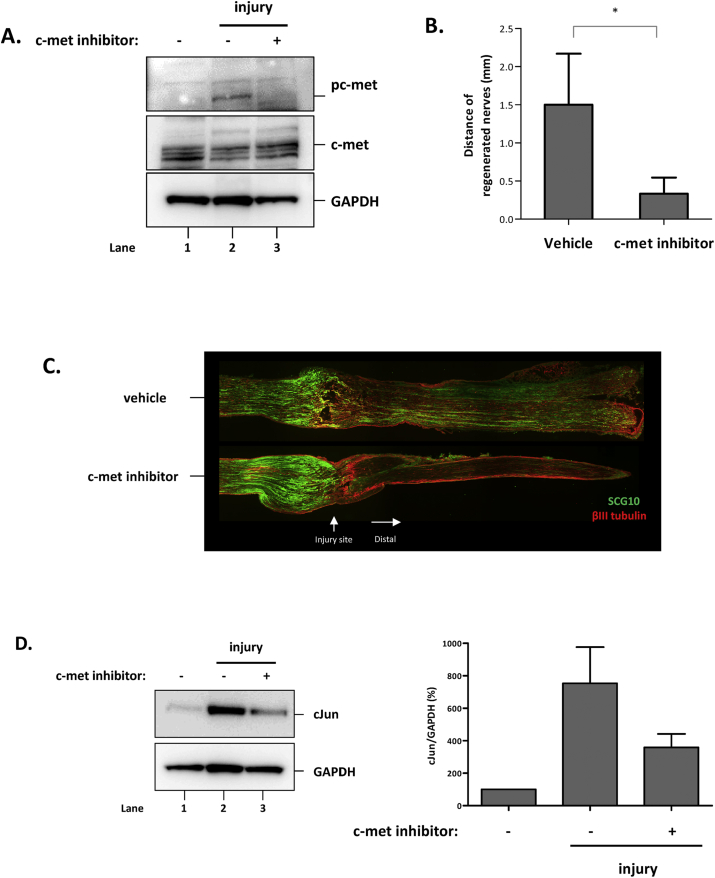
Effect of PHA665752 in the nerve crush mouse model. A) Effect on activated c-met. Injured nerves were harvested by day 3, followed by Western blot using antibodies specific to phosphorylated c-met, c-met, and GAPDH. B) Effect in the nerve pinch test. After 5 days of injury, the distance between the crush and pinch-responsive site was measured. Values are presented as mean ± SEM. For statistical analysis, we performed unpaired *t*-test. *p < 0.05. C) Effect on axon regeneration. Injured nerves from the injury site to distal region were isolated 3 days after injury and subjected to IHC using antibodies specific to SCG10 (in green) and βIII tubulin (in red). D) Effect on cJun expression in DRGs. DRGs were harvested 3 days after injury and then analyzed by Western blot using antibodies specific to cJun and GAPDH. (For interpretation of the references to colour in this figure legend, the reader is referred to the Web version of this article.)

The functional involvement of HGF/c-met pathway was tested in the nerve crush mouse model using PHA665752. After nerve crush injury, mice were treated with PHA665752 around the injury site using surgifoam. The level of phosphorylated c-met was increased after nerve injury, but was reduced to background levels by local treatment of the injury site with PHA665752. This result shows that the procedure involving surgifoam with PHA665752 could effectively block c-met phosphorylation (Fig. 3A).

Next, the sciatic nerve pinch test was performed to assess the effect of HGF/c-met pathway on axon regeneration [16]. Five days after injury, the distance between the injury and distal sites where mouse show withdrawal reflex was measured. In control mice, the distance was 1.66 ± 0.31 mm. When c-met inhibitor was applied locally to the injury site, the length was reduced by 65% to 0.58 ± 0.27 mm (Fig. 3B). Histological analysis also confirmed the inhibition of axon regeneration by PHA665752. When the injured nerve was analyzed by staining SCG10 protein, regenerating axons were readily observed at the distal region of the sciatic nerve. When PHA665752 was applied to the injury site, however, SCG10-positive regenerating neurons were hardly seen in distal region (Fig. 3C). Taken together, these data suggested that HGF/c-met pathway played a role(s) in the regeneration process of injured peripheral nerves.

The transcription factor, cJun, has been proposed to be a key player in the nerve regeneration process, and is often used as a maker for regeneration [17]. The cJun level in the DRGs was low in normal mice (Fig. 3D, lane 1), but highly increased by 7.5-fold after nerve injury (lane 2). When PHA665752 was applied, its level was greatly reduced, by 53% (lane 3). This result suggested that one of downstream targets of HGF/c-met pathway might be cJun during the peripheral nerve regeneration.

### 3.4 Effect of intramuscular injection of pCK-HGF-X7 on peripheral nerve regeneration

The nerve crush mouse model was then used to investigate if an exogenous supply of HGF could facilitate axon outgrowth. The half-life of HGF protein is very short, less than 5 min, so the use of recombinant protein is not expected to be an efficient way of delivering HGF. In this study, we used a plasmid DNA designed to express human HGF, pCK-HGF-X7, which has been tested in a variety of clinical studies as well as animal experiments [9,18,19].

**Fig. 4:**
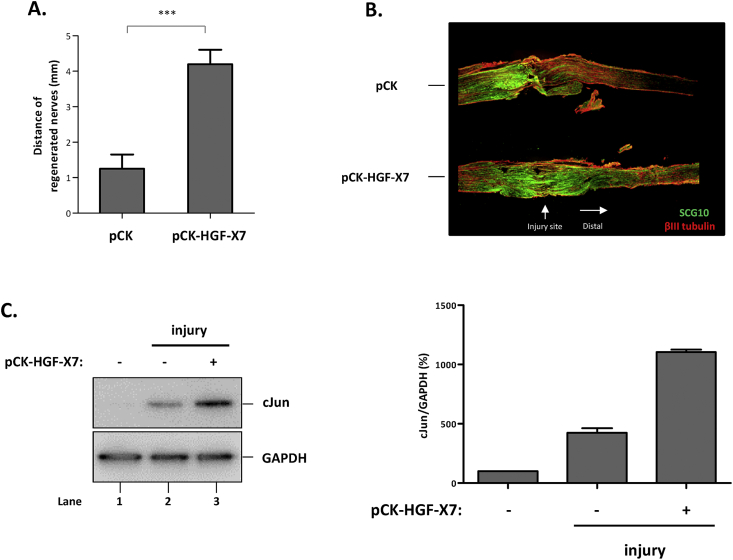
Effects of intramuscular injection of plasmid DNA on nerve regeneration in the nerve crush mouse model. A) Effect in the nerve pinch test. Seven days after injury, the distance between the crush and pinch-responsive sites was measured. Values are shown as mean ± SEM. Data was analyzed by unpaired *t*-test for statistical analysis. ***p < 0.001. B) Effect on axon regeneration. Three days after injury, injured nerves from the injury site to distal region were collected, followed by IHC using antibodies specific to SCG10 (in green) and βIII tubulin (in red). C) Effect on cJun expression in DRGs. DRGs were harvested 7 days after injury followed by Western blot using antibodies specific to cJun and GAPDH. (For interpretation of the references to colour in this figure legend, the reader is referred to the Web version of this article.)

In mice injected with a pCK control vector lacking the HGF sequence, the length of regenerated axons was 1.25 ± 0.40 mm, but this length was highly increased by 3.3-fold, to 4.2 ± 0.41 mm, in mice injected with pCK-HGF-X7 (Fig. 4A). Consistent with this result, the increased length of SCG10-positive axon was also observed in mice injected with an HGF expression vector (Fig. 4B). The injury-mediated cJun induction in the DRGs was further enhanced by 2.6-fold in the group of mice injected with HGF-expressing plasmid DNA (Fig. 4C). Together, these data suggested that additional supply of HGF, in the form of plasmid DNA expression vector, enhanced the regeneration of damaged peripheral nerves by promoting axon outgrowth.

## 4 Discussion

This study investigated the role of HGF/c-met pathway in injured peripheral nerves. In *in vitro* primary eDRGs, axotomy increased the level of HGF expression and also activated c-met. Treatment with PHA665752 hindered the neurite outgrowth, while addition of recombinant HGF proteins enhanced this process. In the *in vivo* nerve crush mouse model, nerve injury significantly increased the level of HGF expression, consequently leading to the activation of c-met receptor in peripheral axons. When PHA665752 was applied locally to the injured nerves, the axon outgrowth process was inhibited and the level of cJun expression in DRGs was also decreased. Finally, exogenously administered HGF by intramuscular injection of HGF-expressing plasmid DNA to mice further accelerated the axon outgrowth and increased the expression of cJun.

For axon regeneration to occur efficiently after injury, two components have to be involved, neuronal intrinsic and non-neuronal extrinsic systems [20]. HGF seems to work through both systems. In this study, data from eDRG cultures indicated that HGF could directly interact with sensory neurons. HGF appears to be one of early proteins upregulated by axon injury, and subsequently interacts with c-met receptors on cells around the injury site. It is interesting that activated c-met receptors in proximal regions were localized in axons. For example, it has been shown that activated molecules such as Erk, STAT3, DLK, and JNK move to the DRGs through microtubules and regulate the expression of RAGs including ATF3, CREB, galanin, and cJun in the cell body [[21], [22], [23], [24], [25], [26], [27]]. However, the upstream factors that regulate these signaling molecules in injury site are largely unknown. In this study, we observed that HGF/c-met pathway was activated in the injured sciatic nerve, but not in the soma, while the expression of cJun in DRGs was regulated under the influence of this signaling. Therefore, the HGF/c-met pathway activated by nerve injury might induce some factor(s) in situ that is used to send a signal to the cell body.

HGF also plays roles through the extrinsic system. We have previously shown that HGF induced upon nerve injury interacts with Schwann cells located in distal region, and activated HGF/c-met pathway increased the proliferation and migration of Schwann cells, thereby accelerating the re-myelination process [9]. Therefore, HGF may be an important integral part of axon regeneration system when peripheral nerves are injured.

Intracellular calcium influx plays important roles in the early stage of peripheral nerve regeneration [3]. Nerve injury triggers calcium influx to induce the expression of regeneration-associated genes such as cJun, CREB, and DLK. In this study, we observed that the HGF/c-met pathway increased the expression of cJun, one of the calcium-related genes, in DRGs. In addition, it has been reported that HGF enhances the intracellular calcium level [28,29]. Therefore, the HGF/c-met pathway might facilitate the peripheral nerve regeneration by upregulating cJun expression through a calcium-dependent manner. It is interesting to note that in patients taking calcium channel blockers such as pregabalin and/or gabapentin used as first-line treatments for diabetic neuropathy, the pain-reducing effect of plasmid DNA designed to express human HGF was significantly reduced in clinical trials [19,30]. It is tantalizing to hypothesize that calcium is necessary for nerve regenerating effects of HGF and thus the use of calcium channel blockers for neuropathic pain may require caution.

In summary, HGF appears to contain multiple bioactivities in the context of peripheral nerve regeneration. It interacts with sensory neurons as well as Schwann cells, two key cell types of the peripheral nervous systems. In addition, HGF showed strong anti-inflammatory activities and analgesic effects by controlling the expressions of inflammatory molecules such as CSF-1 and IL-6 and channel proteins like α2δ1 [18]. Given such a wide spectrum of biological effects and their potential to contribute to the regeneration of damaged nerves, HGF may be an excellent starting point for developing new therapeutics for various clinical conditions resulting from peripheral nerve damage.

## Funding

This work was financially supported by Helixmith Co. Ltd.

## Author statement

**Nayeon Lee**: Conceptualization, Methodology, Formal analysis, Investigation, Writing-Original Draft, Writing-Review&Editing; **Sang Hwan Lee**: Methodology, Investigation, Visualization; **Junghun Lee**: Conceptualization, Methodology, Investigation, Writing-Original Draft, Writing-Review&Editing; **Mi-Young Lee**: Methodology, Investigation, Visualization; **Jaegook Lim**: Validation, Investigation, Resource; **Subin Kim**: Validation, Investigation, Resource; **Sunyoung Kim**: Conceptualization, Writing-Original Draft, Writing-Review&Editing, Supervision

## Declaration of competing interest

The authors declare that they have no known competing financial interests or personal relationships that could have appeared to influence the work reported in this paper.
